# Systemic Lupus Erythematosus Patients Exhibit Reduced Expression of *CLEC16A* Isoforms in Peripheral Leukocytes

**DOI:** 10.3390/ijms160714428

**Published:** 2015-06-25

**Authors:** Rachel C. Y. Tam, Alfred L. H. Lee, Wanling Yang, Chak Sing Lau, Vera S. F. Chan

**Affiliations:** 1 Department of Medicine, Li Ka Shing Faculty of Medicine, University of Hong Kong, Hong Kong, China; E-Mails: racheltam86@gmail.com (R.C.Y.T.); leelh1107@gmail.com (A.L.H.L.); cslau@hku.hk (C.S.L.); 2 Department of Paediatrics and Adolescent Medicine, Li Ka Shing Faculty of Medicine, University of Hong Kong, Hong Kong, China; E-Mail: yangwl@hku.hk

**Keywords:** systemic lupus erythematosus, *CLEC16A*, autoimmunity, spliced variants

## Abstract

Systemic lupus erythematosus (SLE) is a prototypic autoimmune disease with multiple etiological factors. The SLE susceptibility locus on chromosome 16p13 encodes a novel gene *CLEC16A* and its functional relationship with SLE is unclear. This study aimed to investigate the expression correlation of the two major *CLEC16A* spliced transcripts with SLE development. Expressions of the long (V1) and short (V2) *CLEC16A* isoforms in the peripheral blood mononuclear cells (PBMCs) were assayed by quantitative real time PCR and compared between healthy individuals and SLE patients. Correlation of *CLEC16A* isoform expression levels with SLE susceptibility, disease severity and twelve clinical parameters were also evaluated. Full length transcripts of *CLEC16A* V1 and V2 isoforms were readily amplified from PBMCs of healthy controls and patients at varying abundance. Compared with healthy controls (*n* = 86), expression levels of V1 and V2 were significantly reduced by ~two- and four-fold respectively in SLE patients (*n* = 181). The relative V2/V1 ratio was also significantly reduced by approximately two-fold. With regard to SLE disease parameters, only a weak positive correlation was found between *CLEC16A* V1 expression levels and SLE disease activity index (SLEDAI) score. Taken together, *CLEC16A* was found to be a susceptibility factor for SLE, with possible contribution to the development of the disease.

## 1. Introduction

Systemic lupus erythematosus (SLE) is a prototypic autoimmune disease characterized by the loss of tolerance to self-antigens, dysregulated autoreactive T- and B-cell activation, production of autoantibodies (auto-Abs) and perturbed cytokine activities. Predominantly, SLE affects young females of child-bearing age and the chronic systemic inflammation of multiple tissue organs often leads to significant morbidity and mortality [1]. SLE is a complex disease with multiple etiological factors. Genome-wide association studies (GWAS) have revealed genetic risk loci encompassing various immune components related to immune complex and antigen clearance (*ITGAM*, *TREX1*, FcγR genes, complement genes), T-cell activation (*PTPN22*, *TNFSF4*, HLA class II), B-cell signaling (*BANK1*, *BLK*, *PRDM1*) as well as TLR-IFN pathways (*IRF5*, *STAT4*, *IRAK1*) [2]. Remarkably, many novel lupus-susceptibility gene regions, such as the *ATG5-PRDM1* intergenic region, *UBE2L3*, *WDFY4* and *KIAA0350* [3,4,5,6,7], have also been identified. However, the functions of these encoded gene products are still unclear. Of particular interest, the *KIAA0350* locus on chromosome region 16p13 contains a novel gene, *CLEC16A*, of the C-type lectin superfamily.

C-type lectin domain family 16, member A (*CLEC16A*) was first described as an autoimmunity-associated gene in a GWAS, which reported that several of its non-coding single-nucleotide polymorphism (SNP) variants were in strong linkage disequilibrium with type I diabetes in Europeans [8]. Such an association was later replicated in a Chinese Han population [9]. *CLEC16A* was also found to be genetically associated with a number of other autoimmune disorders including multiple sclerosis (MS) [10,11,12], rheumatoid arthritis [12], and Crohn’s disease [13] as well as SLE [3,14]. Thus far, the clinical relevance of the genetic association of *CLEC16A* with SLE remains elusive. C-type lectins, being important innate receptors that shape both the innate and adaptive immune responses, are implicated to play critical roles in the pathogenesis of autoimmune diseases [15,16]. Expression and functional irregularities of several C-type lectins, including mannose receptor, mannose-binding lectin (*MBL*) and dectin-1, have been shown to associate with SLE [17,18,19]. For instance, the low-producing *MBL* genetic variants were shown to be associated with SLE and low serum MBL levels would render individuals for increased risk of SLE development [20]. As a putative C-type lectin, *CLEC16A* may potentially play an important role in SLE pathogenesis. Earlier studies showed that *CLEC16A* could have similar functions as its *Drosophila* ortholog in promoting endosomal trafficking and autophagy [21,22]. Evidence from murine models of diabetes also supports its functional involvement in autophagy [23,24]. How these functional attributes of *CLEC16A* correlate with SLE remains unclear. Here, in an attempt to investigate the potential contribution of *CLEC16A* to SLE development, we evaluated the expression of two *CLEC16A* spliced transcripts in peripheral leukocytes of SLE patients and healthy individuals. Correlation of *CLEC16A* isoform expression levels with SLE susceptibility, disease severity and clinical parameters were also evaluated.

## 2. Results and Discussion

### 2.1. Results

**Figure 1:**
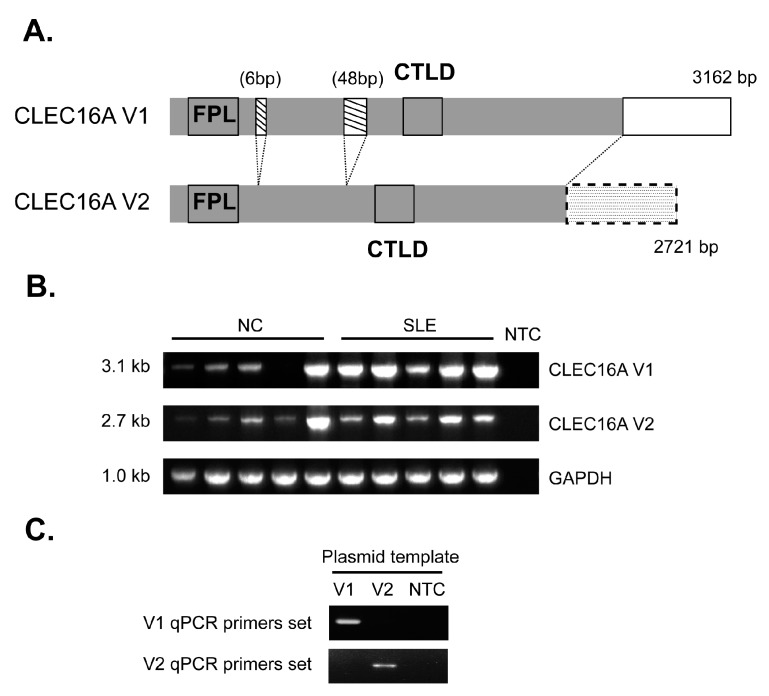
*CLEC16A* expression in peripheral blood mononuclear cells. (**A**) Schematic diagrams of two expressed transcripts of *CLEC16A* showing the predicted FPL and putative C-type lectin-like (CTLD) domains. Grey segments denote identical nucleotide sequences, while the open and dotted segments represent unique sequences in the 3′-end regions of V1 and V2 respectively. Hatched segments represent the 6-bp and the 48-bp sequences observed in V1 but not in V2; (**B**) Full length mRNA transcripts of V1 and V2 could be amplified by RT-PCR from PBMCs of healthy normal controls (NC) and lupus (SLE) patients. Five representative samples from each group are shown. GAPDH was used as an internal control. NTC represents no template control; and (**C**) Specificity of qPCR primer sets for V1 and V2 was tested by conventional PCR using V1 and V2 plasmids as templates. No cross amplification was found.

The human *CLEC16A* gene has been reported to give rise to three alternatively spliced mRNA transcript variants. The longer *CLEC16A* V1 isoform (referred as V1 hereafter) is the canonical isoform expressing all 24 exons, while *CLEC16A* V2 isoform (referred as V2) contains only 21 exons. Apart from isoform 3, which comprises only four exons, V1 and V2 are predicted to encode functional proteins. Sequences of V1 and V2 were thus retrieved from the NCBI Reference sequence database for comparison. The open reading frame (ORF) of V1 (accession no.: NM_015226) comprises 3162 bp which encodes a protein containing a highly conserved FPL domain at the 5′-end, and a putative C-type lectin-like domain (CTLD) in the middle region (Figure 1A). The FPL domain comprises approximately 150 residues that are shared by a family of proteins of unknown function. The ORF of V2 (accession no.: NM_001243403.1) is 441 bp shorter than V1. Sequence alignment analyses revealed the absence of a 6-bp and a 48-bp in-frame segments in V2, and the ~500-bp at the 3′-end of V1 and ~130-bp of V2 were largely non-overlapping (Figure 1A).

No known functional motifs have been predicted in the unique 3′-end regions of both V1 and V2, and it is unclear if the protein products of V1 and V2 may have different functions. It is also not known if both *CLEC16A* isoforms are expressed by immune cells. We therefore first tested the presence of full length transcripts of these two variants. By conventional RT-PCR, full length transcripts of V1 and V2 were readily amplified from peripheral blood mononuclear cells (PBMCs) of healthy controls and SLE patients (Figure 1B). Albeit not quantitatively measured, the relative abundance of V1 and V2 appeared to differ within and between PBMC samples (Figure 1B). Thus, a quantitative PCR (qPCR) assay was designed to compare the expression levels of V1, V2 and their relative expression ratio between normal controls and SLE cases. Specific qPCR primer sets were designed targeting the unique 3′-terminal regions of V1 and V2. These primer sets were highly specific as validated by the absence of PCR cross-amplification of plasmid templates carrying the other isoforms (Figure 1C).

A total of 190 SLE patients were enrolled. Their demographic and clinical characteristics were summarized in Table 1. Blood samples from 86 age-matched normal healthy female controls (NC, median age 43, range 21–67) were obtained.

**Table 1:** Demographics and clinical parameters ^#^ of SLE patients recruited in the study.

Parameter	Value	Unit
Age	46 (20–79)	median years (range)
Female:male	181:9	number
SLE duration	17.5 (1–40)	median years (range)
Malar rash	11.5	%
Discoid rash	0	%
Photosensitivity	0	%
Oral ulcers	5.7	%
Arthritis	3.1	%
Serositis	0.5	%
Renal disorder	14.6	%
Neurological disorder	0	%
Hematological disorder	10.4	%
Immunological disorder	43.2	%
Antinuclear factor	3.1	%
SLEDAI score	3.4 ± 3.7	mean ± SD
Anti-dsDNA	63.5 ± 80.7	mean ± SD
C3 level	82.0 ± 25.0	mean ± SD
C4 level	16.5 ± 7.9	mean ± SD
CRP level	0.5 ± 0.5	mean ± SD

^#^ Clinical parameters recorded at the time of blood sampling for *CLEC16A* expression evaluation.

**Figure 2:**
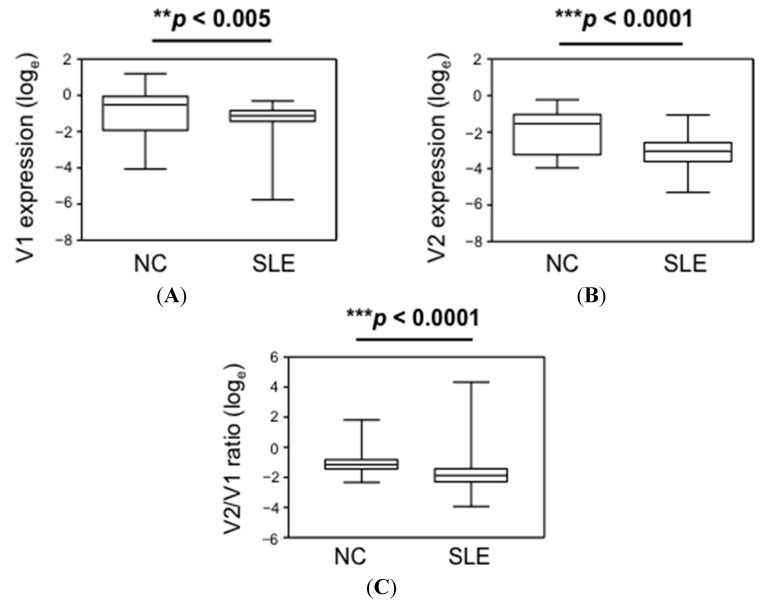
Reduced expression of *CLEC16A* isoforms in PBMCs of SLE patients. Box and whisker plots comparing the expression of (**A**) V1; (**B**) V2; and (**C**) V2/V1 ratio between normal controls (NC, *n* = 86) and SLE patients (SLE, *n* = 181). V1 and V2 expressions were determined by qPCR and normalized with that of β-actin. Data are shown in arbitrary unit in log_e_ scale. *p*-values were calculated using the Mann-Whitney *U*-test.

Expression levels of V1 and V2 in PBMCs were determined by qPCR and normalized with β-actin. Because the healthy control group comprised females only, we compared them with the 181 females in the SLE patient group (SLE, median age 46, range 21–79). Results showed that V1 was the dominant isoform and expressed more abundantly than V2 in both NC and SLE (Table 2). Comparing NC and SLE, expression levels of V1 and V2 were reduced by ~2- and 4-fold respectively in SLE patients. The relative V2/V1 ratio was also significantly reduced (Figure 2).

**Table 2:** *CLEC16A* isoform expression ^#^ in PBMCs of healthy controls and SLE patients.

Group	*CLEC16A* V1	*CLEC16A* V2	*CLEC16A* V2/V1 Ratio
NC	0.61 (0.02–3.31) ^#^	0.22 (0.02–0.79)	0.32 (0.10–6.1)
SLE	0.33 (0.003–0.74)	0.05 (0.005–0.35)	0.15 (0.02–76.05)
*p*-value *	<0.005	<0.0001	<0.0001

^#^ Expression was determined by qPCR and normalized with the expression of the house-keeping gene β-actin. Numbers shown are median (range) in arbitrary unit; * *p*-value was calculated using Mann-Whitney *U*-test comparing NC *vs.* SLE.

**Figure 3:**
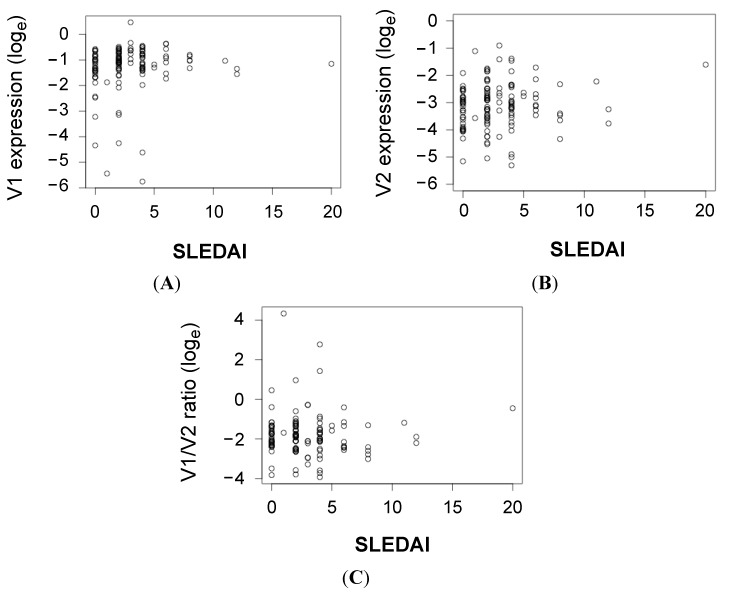
Limited correlation of *CLEC16A* expression with SLE disease severity. Scatter plots showing expression of (**A**) V1; (**B**) V2; and (**C**) V2/V1 ratio against SLE disease activity index (SLEDAI) of 190 SLE patients. Spearman’s Correlation analysis was performed (*Rho* = 0.18, *p* = 0.04 for V1; *Rho* = 0.12, *p* = 0.17 for V2; *Rho* = −0.05, *p* = 0.60 for V2/V1).

Next, we examined if the expression of *CLEC16A* isoforms were correlated with SLE disease severity as reflected in the SLE disease activity index (SLEDAI) score. Figure 3 shows that the majority of SLE patients in this study cohort (>80%) had inactive disease with SLEDAI scores ≤5. Spearman’s correlation analyses revealed V1 expression had a significant but weak positive correlation with SLEDAI score (*Rho* = 0.18, *p* = 0.04 for V1) while no statistical significance was reached for V2 and V2/V1 ratio (*Rho* = 0.12, *p* = 0.17 for V2; *Rho* = −0.05, *p* = 0.60 for V2/V1). The SLEDAI score only reflected disease activity at the time of blood sampling, we therefore also examined expression correlation with the adjusted mean SLEDAI [25] over a two-year period prior to blood sample collection. However, no significant correlation was observed in both isoforms (data not shown). As the SLEDAI is a summation score of various clinical parameters, 12 individual SLE clinical parameters were further evaluated for association with the expressions of *CLEC16A* isoforms. Individually, a significant inverse correlation was observed between V1 expression and leukocyte count, and between V2 expression with C3 level and leukocyte count (Table 3). However, after Bonferroni adjustment for multiple statistical testing, the expressions of *CLEC16A* isoforms did not show any significant correlation with these SLE parameters.

**Table 3:** Correlation analyses of expressions of *CLEC16A* isoforms with twelve clinical parameters in SLE patients.

Clinical Parameter	*CLEC16A* V1	*CLEC16A* V2				*CLEC16A* V2/V1			
	*Rho* *	*p* **	*Rho*	*p*	*Rho*	*p*
Anti-dsDNA titer	0.09	0.32	0.17	0.057	0.10	0.29
C3 level	−0.06	0.53	−0.23	0.0088	−0.18	0.042
C4 level	−0.08	0.40	−0.17	0.053	−0.12	0.18
CRP level	−0.11	0.24	−0.07	0.43	−0.07	0.43
Leukocyte count	−0.24	0.0077	−0.23	0.0087	−0.04	0.68
Hemoglobin count	0.07	0.45	−0.06	0.51	−0.05	0.57
Platelet count	−0.19	0.035	−0.12	0.19	0.08	0.37
Neutrophil count	−0.16	0.076	−0.13	0.14	−0.01	0.95
Lymphocyte count	−0.17	0.050	−0.16	0.077	−0.01	0.95
IgG titer	−0.04	0.70	0.04	0.70	0.01	0.95
IgA titer	0.02	0.83	0.05	0.57	0.02	0.86
IgM titer	−0.12	0.18	−0.14	0.13	0.07	0.46

* Correlation coefficient *Rho* and *p*-value were calculated using Spearman’s correlation test; ** The Bonferroni-adjusted threshold *p*-value is 0.004 for multiple testing (*n* = 12).

### 2.2. Discussion

It is herein shown that the two isoforms of *CLEC16A* are highly expressed in PBMCs of both healthy individuals and SLE patients. Compared with healthy controls, SLE patients presented significantly lower expressions of both V1 and V2, as well as an overall lower V2/V1 expression ratio. Expression of V1 seemed to weakly correlate with SLEDAI scores, however, when the adjusted mean SLEDAI was considered, which was a better reflection of their overall disease severity over two years’ time, no correlation was observed with the expressions of V1, V2 and V2/V1 ratio. Correlation analyses against twelve relevant clinical parameters were also conducted, yet, no significant correlation was observed.

Previous studies have reported the expression of *CLEC16A* in mammalian cells. Public microarray expression datasets and other studies have revealed high *CLEC16A* expression in various immune cell types, particularly B cells, NK cells, dendritic cells, myeloid cells as well as in brain tissues including spinal cord, pineal gland and astrocytes [26,27,28]. Few *CLEC16A* expression correlation studies have been performed, and most of them were conducted in relation to disease-associated genotypes. In the context of MS, a higher relative expression of the *CLEC16A* short and long transcripts (*i.e.*, V2/V1 ratio) in thymic tissues was found significantly associated with the AA risk allele of *CLEC16A* rs12708716 genotype. The absence of this expression correlation in peripheral blood suggests that there may be a thymus- or cell-specific splice regulation for *CLEC16A* [29]. On the other hand, expression association was not observed with two other MS-related SNPs rs6498169 and rs7206912, irrespective of thymus or peripheral blood cells analyzed [29,30]. Recently, a study also reported a significant two-fold increase in total *CLEC16A* transcript in PBMCs of MS patients when compared with normal controls, but the relative expression of the long and short isoforms was not addressed [31]. In type I diabetes, the risk allele at rs12708716 was found correlated with a lower *CLEC16A* expression in human pancreatic β-islet cells. However, no expression comparison was performed between healthy individuals and diabetic patients [23]. To date, apart from the current study, no similar expression correlation investigation has been reported for SLE, whether in association with SLE-related SNPs or with the disease itself. Interestingly, the MS-related rs12708716 SNP is also associated with SLE [3], and the risk AA allele was found in correlation with a higher *CLEC16A* V2/V1 ratio in thymi, but not in peripheral blood cells [29]. Here, a significant reduction in V2/V1 expression ratio in PBMCs of SLE patients was observed instead (Table 2 and Figure 2). Whether this differential expression of *CLEC16A* isoforms is associated with other SLE-related SNPs, or due to tissue-specific splice regulation requires further investigation. However, when compared with MS and type I diabetes, SLE is far more complex in both the etiologies and clinical manifestations. As such, the expression of *CLEC16A* in SLE is likely to be regulated differently according to its contribution to pathogenesis.

Earlier studies in *Drosophila* suggest plausible functional involvement of *CLEC16A* in endosome maturation, trafficking and in promoting autophagy [21,22,32]. In mammalian cells and depending on the cell type, *CLEC16A* expression was localized to different vesicular compartments including endoplasmic reticulum [33], MHC class II vesicles [31] and endolysosomes [23]. *In vitro* knockdown of *CLEC16A* in antigen presenting cells showed severe impairment in cytoplasmic distribution and trafficking of MHC class II endosomal vesicles [31], implicating its critical involvement in antigen presentation. On the other hand, a recent study has revealed the role of *CLEC16A* in promoting type I diabetes via a special form of autophagy–mitophagy–the autophagic removal of mitochondria. Specific functional deficiency of *CLEC16A* in pancreatic β-cells promoted the development of diabetes in mice through the impairment of insulin production as a result of reduced mitophagy and oxygen consumption [23]. Taken together, *CLEC16A* may play important roles in MHC class II antigen presentation as well as autophagy. Intriguingly, HLA-DR is the most significant genetic factor that predisposes SLE development and ATG5, one of the key autophagy-related proteins, is also associated with SLE susceptibility [3]. How *CLEC16A* interacts with these pathways in SLE is still unclear. We have performed *CLEC16A* over-expression and knockdown experiments and our data suggest an inhibitory function of *CLEC16A* in autophagy induction (manuscript in preparation). Hence, the reduced expression of *CLEC16A* isoforms observed in SLE PBMCs may cause enhanced autophagic activities. Indeed, T cells from SLE patients and lupus mice have been shown to contain more autophagic compartments than their corresponding non-lupus control T cells [34]. Further investigations are thus warranted to explore the functional contribution of *CLEC16A* in SLE pathogenesis.

In correlation analyses of clinical parameters, paradoxically, a positive correlation was observed between V1 expression and SLEDAI score. This should be interpreted with caution because the statistical data suggested that this was a weak correlation (small correlation coefficient, *Rho* = 0.18) with marginal significance (*p* = 0.04). It is debatable whether this accurately reflects a true correlation of *CLEC16A* expression with SLE disease severity. The adjusted mean SLEDAI score is a better indication of the overall disease severity over a period of time, but there was no correlation with V1 or V2 expression. Furthermore, *CLEC16A* expression could be up-regulated upon lipopolysaccharides stimulation [28], and it is possible that *CLEC16A* expression may fluctuate in SLE patients because of other non-SLE factors such as microbial infection. Despite being a disease susceptibility factor, *CLEC16A* expression levels in PBMCs did not show any significant correlation with the 12 clinical parameters analyzed (Table 3). Similar observations have been reported in *MBL* polymorphism in lupus patients. Individuals harboring the low-producing *MBL* genotypes were shown to have increased risk of developing lupus but SLE patients showed fluctuating levels of serum *MBL* during the course of disease without any significant correlation with disease parameters [35]. Disease susceptibility and disease progression can be influenced by shared or unique predisposing factors. *CLEC16A* may function as a disease predisposition factor but not a disease modifier, and thus showing disparate association with SLE susceptibility and disease characteristics.

There were two limitations in this study that could also contribute to the lack of association with SLE disease parameters. First, a majority of the patients enrolled in this study were relatively stable with inactive disease. More than 80% of them had SLEDAI scores ≤5, and active patients were under-represented to reliably reflect association with disease severity, if any. Secondly, *CLEC16A* expression was evaluated in bulk PBMCs with varying proportion of T cells, B cells, NK cells and monocytes. All these major immune cell types were reported to express *CLEC16A*, however, which population(s) predominantly express(es) *CLEC16A* is still unknown. Furthermore, the functions of *CLEC16A*, and hence the possible changes in expression levels in different cell populations during disease progression, are unclear. Thus, in a follow-up study to relate *CLEC16A* expressions with SLE clinical manifestations, it would be meaningful to longitudinally examine *CLEC16A* expressions in purified cell populations (e.g., B cells) in patients, and correlate with SLEDAI scores and clinical parameters over a period of time. This would be helpful to directly relate *CLEC16A* expressions and disease severity, if any, and to pinpoint the sub-phenotype(s) of lupus that *CLEC16A* expressions might affect. In addition, relating the changes in expression levels in specific cell population(s) with the presentations of sub-phenotypes would provide clues as to how *CLEC16A* is involved in the progression of SLE.

## 3. Experimental Section

### 3.1. Subjects

SLE patients visiting the Rheumatology clinic at the Hong Kong Queen Mary Hospital (Hong Kong, China) were recruited. Patients enrolled were all of Chinese ethnicity and satisfied the 1997 American College of Rheumatology criteria of SLE classification. Blood samples were collected and patients’ clinical data were used for this study with written informed consent. Ethical approval was reviewed and granted by the Institutional Review Board of the University of Hong Kong and Hospital Authority, Hong Kong West Cluster (HKU/HA HKW IRB). Buffy coats from healthy female volunteer blood donors were obtained from the Hong Kong Red Cross.

### 3.2. Isolation of Peripheral Blood Mononuclear Cells

Human peripheral blood mononuclear cells (PBMCs) were separated by standard density gradient centrifugation using Ficoll-Hypaque Plus (GE Healthcare, Little Chalfont, UK) from whole blood collected into EDTA-tubes or from buffy coats. The purified PBMCs were stained by Trypan blue to confirm cell viability and minimal carry-over of platelets.

### 3.3. Expression Analyses

Total RNA from purified PBMCs was isolated with TRIzol reagent (Invitrogen, Carlsbad, CA, USA). Integrity of RNA was checked by agarose gel electrophoresis. Complementary DNA was synthesized using ThermoScript™ reverse transcriptase (Invitrogen) and oligo (dT)_20_ (Invitrogen) according to the manufacturer’s recommendation. Conventional polymerase chain reaction (PCR) amplification of the full length transcripts of the long isoform of *CLEC16A* (V1, 24 exons, NM_015226), short isoform (V2, 21 exons, NM_001243403.1) and GAPDH were performed with FastStart Taq DNA Polymerase (Roche, Penzberg, Germany). The primer pairs used were as follows: V1-forward: ATGTTTGGCCGCTCGCGGAG, reverse: TGTGTCTTGCCAGTCAGCGGC; V2-forward: ATGTTTGGCCGCTCGCGGAG, reverse: CTAAGAAGCTGGTGCTGCCAG; GAPDH-forward: CATGTTCCAATATGATTCCACCCA, reverse: TTACTCCTTGGAGGCCATGTG. Amplification conditions were as follows: 95 °C, 5 min, followed by 40 cycles of 95 °C for 20 s, 60 °C for 20 s, and 72 °C for 3 min, final extension at 72 °C for 5 min.

Quantitative PCR (qPCR) was performed with KAPA SYBR^®^ FAST qPCR kit (KAPA Biosystems, Wilmington, MA, USA) on StepOnePlus™ Real-Time PCR System (Applied Biosystems^®^, Waltham, MA, USA) to quantify the differential expression levels of V1 and V2 with β-actin as normalization control. The primer pairs used were as follows: V1-forward: CCGTGGCCCAGTGCATAAACC, reverse: GACGATTACCAACTGGTCAGG; V2-forward: GGACCTCCCAATCCAGCCCAC, reverse: ATGAACGAGCTGTGGCGCAGGG; β-actin-forward: CCCAAGGCCAACCGCGAGAAG, reverse: GTCCCGGCCAGCCAGGTCCAG. Amplification conditions: 95 °C, 3 min, followed by 40 cycles of 95 °C for 3 s, 63 °C for 15 s, and 72 °C for 15 s. The three primer pairs were designed and verified to produce amplification of single-sized products with ~200 bp at comparable amplification efficiency. For quantification, known copy number (from 10^2^–10^7^) of plasmids encoding the full length of V1, V2 and β-actin were included in each qPCR run to generate standard curves. Copy number of V1, V2 and β-actin in the cDNA samples were derived from the corresponding standard curves. Expressions of V1 and V2 were normalized with β-actin for analyses and expressed in arbitrary unit using the following formula: (V1 or V2_[copy number]_/β-actin_[copy number]_)/1000.

### 3.4. Statistical Analyses

Statistical analyses were performed using GraphPad Prism version 5.01 (GraphPad Software Inc., La Jolla, CA, USA) and R-3.1.0 (The R Foundation for Statistical Computing, Vienna, Austria). A *p*-value of less than 0.05 was considered statistically significant unless specified. Mann-Whitney *U*-test was used to compare expression difference between patients and controls. Expression correlation with clinical parameters was evaluated using Spearman’s Correlation test.

## 4. Conclusions

Here, we reported the expressions of the two *CLEC16A* transcript isoforms in PBMCs of healthy individuals and SLE patients. The expressions of both isoforms were significantly lowered in SLE patients albeit no clinical parameters correlation was established. Taken together, CLEC16A was found to be a susceptibility factor for SLE, with possible contribution to the development of the disease.
